# Supplementary material for: A Quassinoid Diterpenoid Eurycomanone from Eurycoma longifolia Jack Exerts Anti-Cancer Effect through Autophagy Inhibition
Source: Molecules. 2022 Jul 8;27(14):4398. doi: 10.3390/molecules27144398 (PMC9324291; doi:10.3390/molecules27144398)
Supplement: Supplementary file 1 [file molecules-27-04398-s001.zip › molecules-1742710-supplementary.pdf]

Supplemental Information

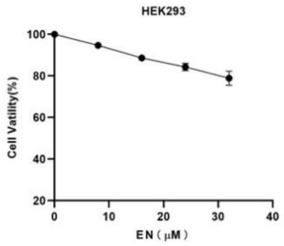

Figure S1. EN inhibits human colon cancer cell proliferation. HEK293 cells were treated with EN (0, 8, 16, 24, 32 μM) for 24 h,

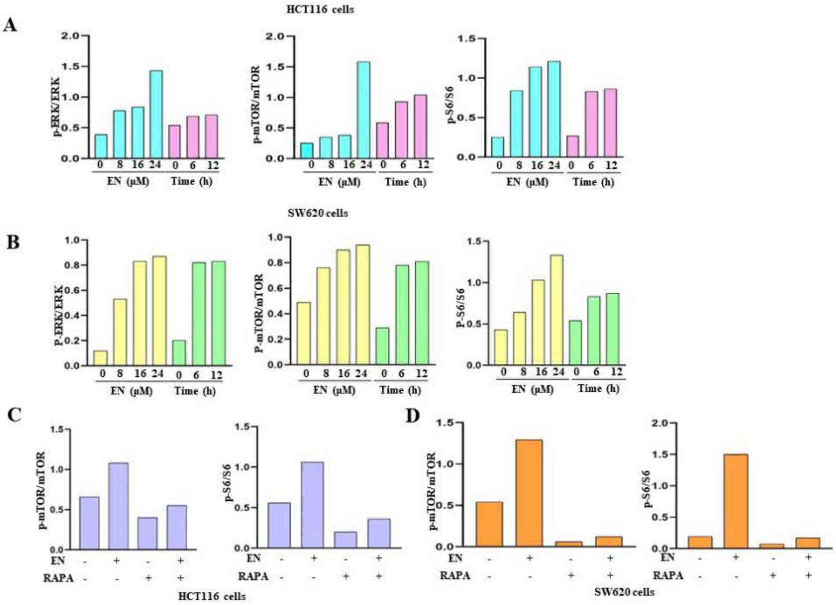

Figure S2. EN activates the mTOR signaling pathway and inhibits autophagy. (A) HCT116 cells were treated with different concentrations of EN (0, 8, 16, 24 μM) and with EN(16μM) for different time (0, 6, 12, 24 h). Western blotting was used to analyze the phosphorylation levels of ERK, mTOR and S6, imageJ was used to calculate the gray scale of western blot strips levels. (B) as in (A), SW620 cells were treated with EN and harvested for western blotting analysis, imageJ was used to calculate the gray scale of western blot strips levels. (C) HCT116 cells were pretreated with rapamycin (200 nM) for 2 h and then continued to be treated with EN (16 μM) for 24 h. Cells were harvested and lysed for western blotting to detect the phosphorylation levels of mTOR and S6, imageJ was used to calculate the gray scale of western blot strips levels. (D) as in (C) SW620 cells were pretreated with rapamycin and EN, imageJ was used to calculate the gray scale of western blot strips levels.
